# Education, Pregnancy Status, and Diet Adherence in Gestational Diabetes: Perceived Burden of Dietary Management

**DOI:** 10.3390/jcm15010340

**Published:** 2026-01-02

**Authors:** Katarzyna Tomczewska, Katarzyna Tomczyk, Małgorzata Kampioni, Witold M. Kędzia, Paweł Rzymski, Małgorzata Kędzia

**Affiliations:** 1 Department of Mother’s and Child’s Health and Minimally Invasive Gynecology, Gynecologic and Obstetrical University Hospital, Poznan University of Medical Sciences, 61-701 Poznan, Poland; 2 Department of Reproduction and Gynecology, Gynecologic and Obstetrical University Hospital, Poznan University of Medical Sciences, 61-701 Poznan, Poland; 3 Student Scientific Association of Department of Reproduction and Gynecology, Poznan University of Medical Sciences, 61-701 Poznan, Poland

**Keywords:** gestational diabetes, quality of life, diet, education

## Abstract

**Background**: Gestational diabetes mellitus (GDM) is one of the most common metabolic complications of pregnancy, and its prevalence continues to rise worldwide. Dietary management is the cornerstone of therapy, yet adherence may impose a substantial everyday burden. This study aimed to assess perceived burden and practical challenges related to following a diabetic diet in women with GDM. **Methods**: A cross-sectional anonymous online questionnaire study was conducted among 109 women with a current or past diagnosis of GDM within the previous five years. The survey addressed self-reported difficulties in maintaining normal blood glucose levels, adherence to a diabetic diet, perceived increases in grocery expenses, time required for meal preparation, dietary preferences, and family attitudes toward the diet. Associations between categorical variables were analyzed using contingency tables and the contingency coefficient. **Results**: Women with insulin-treated GDM (GDM2) reported more difficulties maintaining normal blood glucose levels than women treated with diet and physical activity alone (GDM1) (*p* = 0.014). Educational level was associated with perceived financial burden (*p* = 0.013) and meal preparation time (*p* = 0.003). These patterns likely reflect both differences in economic resources and the extent of dietary changes undertaken, rather than uniform differences in nutritional awareness. Pregnancy status was associated with dietary preferences, as non-pregnant respondents more often reported liking diabetic-diet meals than pregnant respondents (*p* = 0.037). Overall, 53.2% of respondents reported that a diabetic diet made daily functioning more difficult, mainly due to increased time and financial demands. **Conclusions**: Dietary management of GDM is associated with a meaningful perceived burden, especially among women requiring insulin therapy and those facing financial and time constraints. Understanding these subjective challenges may support more individualized dietary counseling and patient-centered care.

## 1. Introduction

Gestational diabetes mellitus (GDM) is currently one of the most common metabolic complications of pregnancy, and its prevalence continues to rise globally [1,2,3,4]. According to the Polish Society of Gynecologists and Obstetricians (PTGiP), GDM affects approximately 5–6% of pregnancies in Europe, although epidemiological trends indicate a steady upward trajectory, particularly in regions with increasing rates of obesity and delayed childbearing [2,5]. GDM typically develops in the second half of pregnancy and is usually asymptomatic, which makes routine screening essential. The diagnostic criteria established by the World Health Organization (WHO) and the International Federation of Gynecology and Obstetrics (FIGO) highlight fasting glucose ≥ 92 mg/dL or post-load abnormalities as sufficient for diagnosis [6,7]. Early detection is critical, as untreated hyperglycemia poses risks such as preeclampsia, polyhydramnios, fetal overgrowth, neonatal hypoglycemia, and long-term metabolic consequences in both mother and child [8,9,10].

The pathophysiology of GDM is multifactorial and largely driven by progressive insulin resistance induced by placental hormones and pregnancy-related metabolic changes [8,10,11]. Dietary habits, maternal body composition, genetic predisposition, and lifestyle further modulate the risk [12,13].

Clinical guidelines consistently emphasize medical nutrition therapy (MNT), glucose self-monitoring, and pregnancy-adapted physical activity as the foundation of treatment; insulin therapy is recommended when glycemic targets are not achieved with lifestyle measures alone [2,5,7,11]. The dietary approach typically prioritizes low glycemic index/load carbohydrate choices, distributed meals, adequate fiber and micronutrients, and individualized caloric goals [9,12,14]. Consumption of low-glycemic-index carbohydrates has been shown to improve maternal weight gain, reduce postprandial glucose excursions, and decrease the likelihood of requiring insulin therapy [9,12]. Furthermore, physical activity adapted to pregnancy—such as walking, stretching, or Pilates—plays an important supportive role in improving insulin sensitivity and glycemic balance [8,11].

While evidence supports the effectiveness of lifestyle interventions, adherence to dietary recommendations may impose substantial practical and psychosocial challenges, including increased food costs, time demands for meal planning and preparation, and family- and work-related constraints [13,14,15,16]. These factors may influence women’s perceived burden during pregnancy and after delivery, especially when combined with glucose monitoring, pregnancy symptoms, and concerns about fetal health [13,14].

Data describing these subjective aspects of dietary management in GDM are limited in Poland. Therefore, the aim of this study was to evaluate the perceived burden and practical challenges of dietary management among women with a current or past diagnosis of GDM. Using an anonymous online questionnaire, we assessed self-reported difficulties in maintaining blood glucose levels, adherence to a diabetic diet, time and financial demands, dietary preferences, and family attitudes toward diabetic meals, in relation to age, education, pregnancy status, parity, and GDM type.

## 2. Materials and Methods

The study was conducted between December 2024 and May 2025 using an online survey distributed via the Google Forms platform (Google LLC, Mountain View, CA, USA) (members of Facebook Pregnancy Diabetes group). Participation was voluntary and anonymous. Respondents provided electronic consent before completing the questionnaire. A proprietary questionnaire consisting of 26 single-choice questions was used. Items covered sociodemographic characteristics (age, education, pregnancy status, parity, sources of knowledge) and self-reported experiences related to GDM management, including:

- Difficulties maintaining normal blood glucose levels;
- Difficulties adhering to a diabetic diet;
- Perceived increased grocery expenses;
- Increased time required for meal preparation;
- Preference/acceptance of diabetic-diet meals;
- Family consumption of meals prepared according to diabetic-diet criteria.

Although some items concerned daily functioning and well-being, the tool was not a validated quality-of-life instrument. Accordingly, the study focuses on perceived burden and practical challenges, rather than standardized quality-of-life outcomes.

### 2.1. Study Group and Inclusion Criteria

The inclusion criterion was a current or previous diagnosis of gestational diabetes mellitus (GDM), no previous DM or additional endocrinological disorders in the medical history, no previous diabetic diet. The time since the previous pregnancy was a maximum of 5 years.

**Figure 1:**
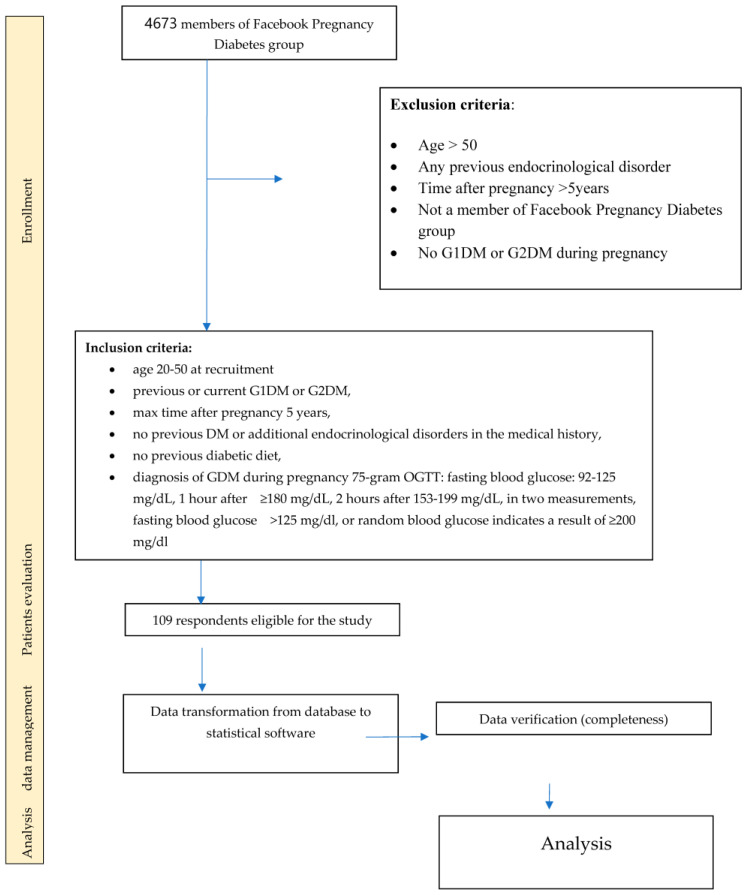
Selection of the study group.

A total of 109 respondents meeting the inclusion criterion participated in the study (Figure 1).

### 2.2. Statistical Analysis

All statistical analyses were performed using IBM SPSS Statistics, version 30.0.0. (IBM, Armonk, New York, NY, USA). Variables were summarized as counts and percentages. Associations between categorical variables were assessed using contingency tables and the contingency coefficient; *p* < 0.05 was considered statistically significant. Not all participants answered every question; therefore, percentages are calculated based on the number of available responses for each item. Given the cross-sectional design, results are interpreted as associations rather than predictors or causal determinants.

## 3. Results

### 3.1. Group Characteristics

The study included 109 respondents. The detailed characteristics of the group are presented in Table 1.

#### 3.1.1. Associations with Pregnancy Status

The analysis of symmetric measures demonstrated that the contingency coefficient was 0.196, with a *p*-value of 0.037. This result indicates a statistically significant relationship between pregnancy status and preferences for diabetic-diet meals. Notably, a substantially higher proportion of non-pregnant respondents reported liking meals that meet the criteria of a diabetic diet (82.1%) compared with pregnant respondents (60.5%) (Table 2).

#### 3.1.2. Associations with Type of GDM Treatment (GDM1 vs. GDM2)

A statistically significant relationship was observed between the type of gestational diabetes and difficulties in maintaining normal blood glucose levels (contingency coefficient = 0.230; *p* = 0.014). Women requiring insulin therapy (GDM2) more frequently reported difficulties compared with women treated with diet and physical activity (GDM1) (Table 3).

#### 3.1.3. Associations with Parity

No statistically significant relationship was found between number of births and time required to prepare meals (*p* = 0.755). The corresponding column percentages illustrating this relationship are presented in Table 4.

#### 3.1.4. Association with Education

(a) Glycemic control

The analysis showed no statistically significant relationship between education level and difficulty maintaining normal blood glucose levels (*p* = 0.070).

(b) Perceived increased grocery expenses

Education level was significantly associated with reporting higher grocery expenses while following a diabetic diet (*p* = 0.013).

The corresponding column percentages are presented in Table 5.

Women with primary and higher education most frequently reported increased expenses, whereas respondents with secondary or vocational education more often reported no additional costs. This pattern should be interpreted cautiously: in women with higher education, greater resources and health literacy may facilitate broader dietary changes (including the purchase of higher-cost or specialized products), while in women with primary education even modest dietary adjustments may represent a substantial financial strain due to limited baseline resources.

(c) Increased time needed to prepare meals

Education level was significantly associated with reporting more time required for meal preparation (*p* = 0.003).

The corresponding column percentages for this relationship are shown in Table 6.

Women with primary and higher education more frequently reported that preparing diabetic-diet meals required additional time. Importantly, this similarity likely reflects different mechanisms: women with higher education may implement more extensive dietary changes requiring planning and cooking from basic ingredients, whereas women with primary education may experience increased complexity and time burden when attempting to adapt traditional, low-budget meals to dietary recommendations under constrained resources.

(d) Preferences for diabetic-diet meals

No statistically significant relationship was found between education level and liking diabetic-diet meals (*p* = 0.207). Elementary education represented 11.9% of the sample (*n* = 13); estimates for this subgroup should be interpreted cautiously due to its size.

The corresponding column percentages for this relationship are shown in Table 7.

(e) Family consumption of diabetic meals

No statistically significant association was found between education level and family members eating the same meals (*p* = 0.113). This result should be interpreted as “no association detected” rather than evidence of no real-world relationship, since family involvement may depend on unmeasured social and economic factors.

The corresponding column percentages and coefficient values are presented in Table 8.

#### 3.1.5. Associations with Age

No statistically significant relationships were observed between age and the analyzed outcomes (difficulties maintaining normal glucose levels, diet adherence, increased expenses, meal preparation time, dietary preferences, family consumption), with all *p*-values ≥ 0.05.

## 4. Discussion

This cross-sectional questionnaire study evaluated the perceived burden and practical challenges associated with dietary management in women with current or past gestational diabetes mellitus (GDM). Several findings reached statistical significance and are therefore of particular clinical relevance.

First, a statistically significant association was observed between the type of GDM and difficulties in maintaining normal blood glucose levels. Women requiring insulin therapy (GDM2) reported significantly more problems with glycemic control compared with women managed with diet and physical activity alone (GDM1). This finding is consistent with established clinical evidence indicating that insulin-treated GDM reflects more advanced metabolic impairment, greater insulin resistance, and higher glycemic variability, all of which increase the complexity of daily self-management and the need for intensified monitoring and support [8,9,11].

Second, education level was significantly associated with both perceived financial burden and time demands related to dietary management. Women with elementary and higher education most frequently reported increased grocery expenses and longer meal preparation times. Importantly, these statistically significant patterns likely reflect different underlying mechanisms rather than similar behavioral or cognitive determinants. Among women with higher education, greater health literacy and financial resources may facilitate more comprehensive implementation of dietary recommendations, including the purchase of specialized or higher-quality foods, which inevitably increases costs and preparation time [11,13,17]. In contrast, among women with elementary education, even limited dietary modifications may constitute a disproportionate burden due to restricted household budgets and time constraints. Therefore, these findings should be interpreted primarily in the context of economic limitations and heterogeneity in baseline resources, rather than as evidence of comparable nutritional awareness across educational groups. Notably, the statistically significant increase in perceived time and financial burden aligns with international literature demonstrating that medical nutrition therapy (MNT), although clinically effective, imposes substantial lifestyle and financial strain on many women with GDM [11,13,17].

Third, pregnancy status showed a statistically significant association with dietary preferences. Pregnant respondents were significantly less likely to report liking meals that meet diabetic-diet criteria compared with non-pregnant respondents. This finding may be explained by pregnancy-related symptoms such as nausea, fatigue, altered taste perception, and food aversions, as well as the psychological stress associated with pregnancy complications and dietary restrictions [16,18,19,20,21]. In contrast, women who were no longer pregnant may experience greater flexibility in food choices, reduced symptom burden, and more opportunity to adapt eating patterns, which may improve acceptance of dietary recommendations [20,21,22].

In contrast, no statistically significant associations were observed for age or parity with any of the analyzed outcomes, including glycemic difficulties, perceived dietary burden, or dietary preferences. These null findings may indicate a true lack of association; however, the limited sample size within specific subgroups may have reduced statistical power to detect smaller effects.

Overall, the statistically significant findings of this study highlight that type of GDM, education level, and pregnancy status are key factors shaping the perceived burden of dietary management in women with GDM. These results underscore the importance of individualized dietary counseling that accounts not only for metabolic severity but also for socioeconomic context and pregnancy-related challenges.

## 5. Limitations

This study has several limitations. Recruitment via a Facebook support group may introduce selection bias toward women who are more digitally engaged or health-motivated than the general GDM population, limiting generalizability. The study was cross-sectional and included women currently pregnant as well as women recalling experiences up to five years after pregnancy, introducing heterogeneity and potential recall bias. The study population is not representative of all women with GDM; therefore, the findings should be interpreted as exploratory and hypothesis-generating.

The questionnaire was proprietary and not a validated quality-of-life instrument; therefore, outcomes reflect perceived burden and practical challenges rather than standardized quality-of-life measures. Finally, diagnosis and treatment type were self-reported and no objective glycemic data (e.g., glucose logs, HbA1c) were collected, which prevents verification of adherence and metabolic outcomes.

## 6. Conclusions

Women with insulin-treated GDM (GDM2) more often reported difficulties maintaining normal blood glucose levels compared with those managed with diet and physical activity alone (GDM1). Education level appeared to influence the perceived financial and time burden of dietary management, suggesting that these differences likely reflect varying baseline resources and constraints rather than uniform disparities in nutritional awareness. Pregnancy status was also associated with dietary preferences, as non-pregnant respondents more frequently expressed liking for diabetic-diet meals than pregnant respondents. In contrast, age and parity showed no significant associations with the outcomes analyzed in this study. Notably, more than half of all respondents indicated that dietary management complicates daily functioning, primarily due to increased time and financial demands.

## Figures and Tables

**Table 1:** Group characteristics (*n* = 109).

Variable	Category	*n*	%
Age (years)	20–30	51	46.8
	30–40	50	45.9
	>40	8	7.3
Education level	Higher education	71	65.1
	High school education	25	22.9
	Elementary education	13	11.9
Pregnant at the time of the survey	yes	81	74.3
	no	28	25.7
Occurrence of gestational diabetes	Current occurrence	62	56.9
	Past occurrence	28	25.7
	Past and current occurrence	19	17.4
Trimester of diagnosis	First trimester	58	53.2
	Second trimester	44	40.4
	Third trimester	7	6.4
Number of deliveries	1	86	78.9
	≥2	23	21.1

Results According to Variables.

**Table 2:** Pregnancy status and preference for diabetic-diet meals.

Do You Like Diabetic Diet Meals?	Not Pregnant	Pregnant
No	17.9%	39.5%
Yes	82.1%	60.5%

**Table 3:** Difficulty in maintaining normal glucose levels by GDM type.

Do You Have Trouble Maintaining Normal Blood Sugar Levels?	GDM 1 (Diet + Physical Activity)	GDM 2 (Insulin Therapy)
No	56.3%	31.6%
Yes	43.7%	68.4%
Total	100%	100%

**Table 4:** Meal preparation time by parity.

Do You Spend More Time Preparing Meals While Following a Diabetic Diet?	1 Birth	2 Births and More
No	44.2%	47.8%
Yes	55.8%	52.2%
Total	100.0%	100.0%

**Table 5:** Increased grocery expenses by education.

Education	Primary	Secondary	Higher	Vocational
Do you spend more money on groceries when following a diabetic diet?				
No	0.0%	72.0%	39.4%	63.6%
Yes	100.0%	28.0%	60.6%	36.4%
Total	100.0%	100.0%	100.0%	100.0%

**Table 6:** Increased meal preparation time by education.

Education	Primary	Secondary	Higher	Vocational
When following a diabetic diet during pregnancy, do you spend more time preparing meals?				
No	-	68.0%	33.8%	72.7%
Yes	100.0%	32.0%	66.2%	27.3%
Total	100.0%	100.0%	100.0%	100.0%

**Table 7:** Preferences for diabetic-diet meals by education.

Education	Primary	Secondary	Higher	Vocational
Do you like meals that meet diabetic-diet criteria?				
No	100%	28.0%	35.2%	27.3%
Yes	-	72.0%	64.8%	72.7%
Total	100.0%	100.0%	100.0%	100.0%

**Table 8:** Family consumption of diabetic meals by education.

Education	Primary	Secondary	Higher	Vocational
Does your family also eat the meals you prepare for yourself while on diabetic diet?				
No	100%	24.0%	42.3%	45.5%
Yes	-	76.0%	57.7%	54.5%
Total	100.0%	100.0%	100.0%	100.0%

## Data Availability

The original data presented in the study are openly available.
